# Awareness and Attitudes of the Saudi Population Towards Bariatric Surgery in Saudi Arabia

**DOI:** 10.3390/healthcare12242528

**Published:** 2024-12-13

**Authors:** Baraa Alghalyini, Abdul Rehman Zia Zaidi, Zainudheen Faroog, Mohamad Aljejakli, Najib Fares Garad, Muaadh Shariff Muhammad Nyroze, Muhammad Usaid Ejaz, Mustafa Khedr, Mohamed Yousif, MSM Abrar Fuad Khan, Abdulghaffar Khateeb

**Affiliations:** 1 Department of Family & Community Medicine, College of Medicine, Alfaisal University, Riyadh 11533, Saudi Arabia; balghalyini@alfaisal.edu; 2 College of Medicine, Alfaisal University, Riyadh 11533, Saudi Arabia; zfaroog@alfaisal.edu (Z.F.); maljejakli@alfaisal.edu (M.A.); ngarad@alfaisal.edu (N.F.G.); mnyroze@alfaisal.edu (M.S.M.N.); mejaz@alfaisal.edu (M.U.E.); mukhedr@alfaisal.edu (M.K.); mmyousif@alfaisal.edu (M.Y.); mafkhan@alfaisal.edu (M.A.F.K.); akhateeb@alfaisal.edu (A.K.)

**Keywords:** bariatric surgery, obesity, Saudi Arabia, knowledge, attitudes

## Abstract

**Background**: Obesity is a growing public health concern worldwide, and Saudi Arabia faces rising rates of obesity-related conditions, including diabetes and cardiovascular diseases. Bariatric surgery is a recognized treatment for severe obesity, offering significant health benefits. This study evaluates the Saudi population’s awareness and attitudes towards bariatric surgery, examining knowledge levels, perceptions, and potential barriers. **Methods**: A cross-sectional survey was conducted among adults in Riyadh using convenience and snowball sampling. The survey included demographic data, awareness of obesity and bariatric surgery, attitudes towards the procedure, perceived barriers, and willingness to consider surgery. Data were analyzed using descriptive statistics and logistic regression. **Results**: Among 313 participants (mean BMI 26.98 kg/m^2^), 41.9% identified obesity as a disease, and 48.9% recognized the effectiveness of bariatric surgery for severe obesity. Knowledge scores averaged 4.08 (out of 5) for obesity and 3.28 (out of 6) for bariatric surgery. Significant associations were found between bariatric surgery knowledge and gender (*p* < 0.001), BMI (*p* = 0.004), income (*p* = 0.025), and educational level (*p* = 0.001). While 71.2% would not consider surgery, misconceptions about risks, stigma, and cost remain common barriers. **Conclusions**: Although awareness of bariatric surgery in Saudi Arabia is moderate, misconceptions about safety and financial constraints persist. Public education campaigns are necessary to dispel myths, improve understanding, and promote bariatric surgery as a viable treatment option for severe obesity. Expanding insurance coverage and financial support may also enhance accessibility and adoption of this life-saving intervention.

## 1. Introduction

Obesity, defined as a body mass index (BMI) of 30 kg/m^2^ or higher, represents a global public health crisis with far-reaching consequences [1]. It is a major risk factor for non-communicable diseases, including diabetes, cardiovascular disorders, and certain cancers, all of which contribute to reduced life expectancy and quality of life [2]. Globally, the prevalence of obesity has doubled since 1980, and projections indicate that over 51% of the global population will be overweight or obese by 2035 [3]. The World Health Organization (WHO) has declared obesity a global epidemic due to its widespread prevalence and severe impact on health systems [4].

In the Arab region, obesity prevalence is particularly concerning, with studies reporting rates between 66% and 75% of adults being overweight or obese [5]. Cultural and environmental factors, such as sedentary lifestyles, high-calorie diets, and urbanization, have contributed to these alarming figures [6]. Saudi Arabia exemplifies this trend, where more than 50% of the population is classified as overweight or obese [7]. Recent studies estimate that approximately 68.2% of adults in Saudi Arabia are overweight, with 33.7% meeting the criteria for obesity [8]. These figures surpass global averages, underscoring the urgent need for effective interventions [9].

While lifestyle modifications, including diet and exercise, remain the cornerstone of obesity management, these approaches are often insufficient for individuals with severe obesity (BMI ≥ 40 kg/m^2^) or obesity-related comorbidities (BMI ≥ 35 kg/m^2^ with conditions such as type 2 diabetes) [10]. Bariatric surgery, a procedure that modifies the digestive system to facilitate significant weight loss, has emerged as a highly effective treatment for severe obesity [11]. Studies show that bariatric surgery not only helps individuals lose 20–40 kg within a decade but also resolves comorbid conditions such as diabetes and hypertension, reducing the risk of mortality by over 40% [12,13].

Saudi Arabia has one of the highest rates of bariatric surgery per capita worldwide, with over 27,000 procedures performed annually as of 2019 [14]. Despite its growing popularity, misconceptions about bariatric surgery persist, including concerns about safety, stigma, and financial barriers [15]. Awareness of the procedure, its eligibility criteria, and its long-term benefits is unevenly distributed across the population, with younger, educated individuals showing better knowledge [16,17].

There is limited research on the public’s understanding of bariatric surgery in Saudi Arabia, despite its crucial role in addressing severe obesity. Inadequate knowledge and persistent myths about the procedure hinder its acceptance and appropriate utilization [18]. This study aims to assess the awareness, attitudes, and willingness of the Saudi population towards bariatric surgery, identifying barriers and facilitators to its adoption. The findings from this research will inform targeted educational campaigns and policy measures to promote bariatric surgery as a viable solution for managing severe obesity in the region.

## 2. Materials and Methods

### 2.1. Study Design

This study employed a descriptive cross-sectional design to assess the awareness, attitudes, and willingness of the Saudi population toward bariatric surgery. The cross-sectional approach was chosen as it enables the collection of data from a large sample at a single point in time, making it an efficient method to capture population-level awareness and perceptions. Data collection was conducted from June 2023 to January 2024.

### 2.2. Sampling, Participants, and Data Collection

Participants were recruited using a combination of in-person and online methods to ensure diverse representation. In-person recruitment occurred in public settings such as community centers, shopping malls, libraries, and fitness clubs, providing access to individuals from various social and demographic backgrounds. An anonymous online survey was distributed via widely used social media platforms, including WhatsApp, Facebook, and Twitter. The online component employed the snowball sampling technique, encouraging initial participants to share the survey link within their networks to expand the sample size.

Convenience sampling was utilized due to its practicality in accessing readily available populations; however, this approach inherently limits generalizability. Efforts were made to mitigate this limitation by targeting a variety of urban and semi-urban areas, as well as a diverse range of social media platforms, to capture a broad cross-section of the population.

Participants were eligible if they were adults (aged ≥ 18 years) residing in Saudi Arabia. Individuals younger than 18 years, non-residents, or those with severe language or cognitive impairments were excluded. Prior to participation, all individuals provided electronic informed consent after being presented with a brief overview of the study objectives, ethical considerations, and the voluntary nature of their participation.

### 2.3. The Survey Instrument

A structured questionnaire was adapted from previously published studies focusing on bariatric surgery awareness and knowledge in Saudi Arabia [17,19,20,21]. The questionnaire was designed to capture participants’ demographic characteristics, awareness of obesity and bariatric surgery, attitudes towards these topics, perceived barriers, and willingness to consider bariatric surgery. It consisted of five sections with thirty-three close-ended questions and one optional open-ended question for general comments: (1) demographics, (2) awareness of obesity, (3) awareness of bariatric surgery, (4) attitudes toward obesity, and (5) attitudes toward bariatric surgery.

To ensure content validity, the questionnaire underwent review by an expert panel comprising metabolic doctors, bariatric medicine physicians, bariatric surgeons, and specialists in family and community medicine and public health. A pilot study involving 35 participants representative of the target population was conducted to assess for clarity, relevance, and the average time required for completion. Based on the feedback, minor modifications were made, and an Arabic version of the questionnaire was developed to address language preferences and improve accessibility.

The finalized questionnaire ensured alignment with the study objectives, facilitating comprehensive data collection on knowledge, attitudes, and willingness regarding bariatric surgery.

### 2.4. Sample Calculation

The sample size was calculated using Cochran’s Sample Size Formula, which is widely used for estimating proportions in large populations. Assuming a 95% confidence level (Z = 1.96), a 5% margin of error (e = 0.05), and an estimated population proportion (p) of 50% to maximize variability, the minimum required sample size was determined as follows:

n_0_ = (Z^2^ × p × q)/e^2^

n_0_ = (1.96^2^ × 0.5 × 0.5)/0.05^2^

n_0_ = 385

To account for potential nonresponses and incomplete data, the target sample size was increased to 485 participants. Ultimately, 571 individuals participated in the survey, but 258 were excluded due to incomplete responses, resulting in a final sample size of 313 participants. While the achieved sample size is below the initial target, it remains sufficient for descriptive analyses, albeit with a reduced power to detect smaller effects in inferential statistics.

The use of Cochran’s formula was justified given the absence of prior prevalence data on bariatric surgery awareness in Saudi Arabia, necessitating a conservative approach to ensure representativeness.

### 2.5. Statistical Data Analysis

Data were entered into Microsoft Excel and cleaned to address any inconsistencies or missing responses. Records with incomplete or invalid data were excluded from the analysis. Statistical analyses were performed using SPSS version 28 (Statistical Package for the Social Sciences, IBM Corp., Armonk, NY, USA).

Descriptive statistics were used to summarize demographic characteristics, awareness levels, and attitudes, and the results were presented as frequencies, percentages, means, and standard deviations (SDs). Associations between categorical variables (e.g., gender and educational level) and knowledge or attitudes were assessed using Chi-square tests. For continuous variables, independent sample *t*-tests and ANOVA were employed, where applicable, to compare means across groups.

Multiple-response dichotomy analysis was used for questions allowing multiple answers, and multivariable logistic regression analysis was conducted to identify factors independently associated with knowledge on and attitudes toward obesity and bariatric surgery. Predictor variables in the regression models included demographic factors such as gender, age, educational level, BMI, and income.

A *p*-value of <0.05 was used as the threshold for statistical significance, consistent with conventional standards in biomedical research. This choice balances the need to minimize type I errors while maintaining sensitivity to detect meaningful associations.

### 2.6. Ethical Considerations

This study was conducted in full compliance with the ethical standards outlined in the Declaration of Helsinki. Ethical approval was obtained from the Institutional Review Board (IRB) of Alfaisal University (IRB-20211) on 2 April 2023. Participants were informed of this study’s purpose, objectives, and ethical safeguards through an introductory section accompanying the survey. This section emphasized their right to withdraw at any stage without penalty and assured them of the confidentiality of their responses.

Informed consent was obtained electronically before participation, and the survey was designed to ensure anonymity by not collecting any personally identifiable information. Data were stored securely on encrypted devices accessible only to the research team. Additionally, participants were given the option to skip questions they were uncomfortable answering, further supporting their autonomy.

Measures were implemented to protect participants’ privacy during the data collection process, particularly for in-person recruitment in public settings, where sensitive questions were handled discreetly. For online surveys, access to the survey link was controlled to prevent unauthorized entries.

## 3. Results

A total of 571 participants from various districts of Saudi Arabia participated in completing the survey. A total of 258 participants were excluded because of missing data, resulting in a final sample size of 313 participants.

### 3.1. Sociodemographic Characteristics

A total of 571 individuals participated in the survey; however, 258 responses were excluded due to missing or incomplete data, resulting in a final sample size of 313 participants. While this falls short of the estimated target sample size of 385 participants calculated using Cochran’s formula, the achieved sample size remains sufficient to provide meaningful descriptive insights into the awareness and attitudes of the Saudi population regarding bariatric surgery. Nevertheless, the shortfall may limit this study’s statistical power, particularly for detecting smaller or more nuanced associations between variables. These findings should therefore be interpreted with appropriate caution.

Table 1 displays the characteristics of a group of 313 participants. Out of all the participants, 53% were male, while 47% were female. The majority of the participants were married, accounting for 59.7% of the group. In terms of monthly income level, 24.9% of the participants were unemployed, and the majority of those who were employed earned between SAR 10,000 and 20,000 per month.

When it comes to educational level, the majority of the participants (50.8%) had a bachelor’s degree, followed by 22% who had a post-graduate degree. The mean height of the group was 168.01 cm, while the mean weight was 76.61 kg. The mean BMI was 26.98 kg/m^2^.

Out of the participants, 71.2% (*n* = 223) would not have bariatric surgery if given the opportunity, 20.1% (*n* = 63) would take the surgery, and 8.6% (*n* = 27) responded with “Doesn’t apply” (Table 2).

Participants’ knowledge on and attitudes toward bariatric surgery, the focus of this study, revealed moderate understanding and mixed perceptions. As shown in Table 3, the mean knowledge score for bariatric surgery was 3.28 (±1.46) out of 6. While 62.9% correctly identified BMI > 40 as an indication for surgery, misconceptions were prevalent: 72.2% incorrectly believed that bariatric procedures carry no risks or complications. In contrast, attitudes toward bariatric surgery were mixed. Table 4 demonstrates that while 48.9% agreed that bariatric surgeries contribute to significant weight loss, 59.7% did not consider surgery as their first choice for weight loss, reflecting hesitations likely influenced by safety concerns (39.6% believe that bariatric surgery can lead to death).

Regarding general knowledge of obesity (Table 3), participants exhibited high levels of awareness, with a mean score of 4.08 (±0.60) out of 5. For example, 92.7% recognized that obesity results from excessive fat deposition, and 97.8% understood the role of a healthy diet in weight management.

The mean score of knowledge about obesity is 4.08 (out of 5) and mean score of knowledge about bariatric surgery is 3.28 (out of 6).

Table 4 presents the attitudes of the participants regarding obesity and bariatric surgery. The data show the number and percentage of participants who strongly disagree, disagree, are neutral, agree, or strongly agree with each statement, as well as the total score and mean with standard deviation. The answers to the attitude questions were coded (1–5), where a higher score reflects a more positive attitude.

Regarding obesity, the data show that the majority of the participants (41.9%) believe that obesity is a disease, while a relatively low percentage of the participants (2.6%) believe that obesity is solely caused by genetics. Additionally, a significant proportion of the participants (30.7%) believe that mental illnesses such as anxiety can lead to obesity.

In terms of bariatric surgery, the data show that the majority of the participants (48.9%) believe that bariatric surgeries contribute to significant weight loss in morbidly obese patients, while a relatively low percentage of the participants (3.5%) believe that bariatric surgeries should be illegal. Moreover, a high percentage of the participants (59.7%) do not consider bariatric surgery as their first choice to lose weight, and a significant proportion of the participants (39.6%) believe that bariatric surgery can lead to death.

Table 5 shows a Pearson correlation coefficient of 0.137 and a *p*-value of 0.015, indicating a significant positive correlation between knowledge about obesity and bariatric surgery. This suggests that participants who have a higher level of knowledge about obesity are more likely to have a higher level of knowledge about bariatric surgery as well. However, based on the value of the correlation coefficient, the correlation between the two knowledge scores is weak.

Table 6 presents a Pearson correlation coefficient of 0.194 and a *p*-value of 0.001, indicating a significant positive correlation between attitudes towards obesity and bariatric surgery. This suggests that participants who have a more positive attitude towards obesity are more likely to have a more positive attitude towards bariatric surgery as well. However, based on the value of the correlation coefficient, the correlation between the two knowledge scores is weak.

### 3.2. Factors Associated with Knowledge on Obesity

Multiple linear regression was performed to study factors associated with high knowledge about obesity. The only variable that shows statistically significant results is monthly income level. Participants who earn less than SAR 5000 have less knowledge as compared to unemployed participants; coefficient = −0.223, *p*-value = 0.047. Participants who earn from SAR 5000 to 9999 have less knowledge as compared to unemployed participants; coefficient = −0.37, *p*-value = 0.010 (Table 7). The multiple linear regression shows that positive attitudes regarding obesity were associated with BMI and monthly income level. Participants who earn less than SAR 5000 have more positive attitudes as compared to unemployed participants; coefficient = 1.13, *p*-value = 0.037. Participants who earn from SAR 10,000 to 20,000 have more positive attitudes as compared to unemployed participants; coefficient = 1.58, *p*-value = 0.018. A higher BMI is associated with more positive attitudes; coefficient = 0.08, *p*-value = 0.010 (Table 8).

### 3.3. Factors Associated with Knowledge on and Attitudes Towards Bariatric Surgery

Multiple linear regression was performed to study factors associated with high knowledge about bariatric surgery. The only variables that show significant results are BMI and gender. Females have more knowledge as compared to males; coefficient = 1.66, *p*-value < 0.001. A higher BMI is associated with higher knowledge; coefficient = 0.04, *p*-value = 0.004 (Table 9).

Regarding factors associated with positive attitudes towards bariatric surgery, the only variables that show significant results are educational level and monthly income level. Participants who earn less than SAR 5000 have more positive attitudes as compared to unemployed participants; coefficient = 2.04, *p*-value = 0.005. Participants who earn from SAR 10,000 to 20,000 have more positive attitudes as compared to unemployed participants; coefficient = 2.01, *p*-value = 0.025. Participants with a high school educational level have more positive attitudes as compared to participants with an educational level lower than high school; coefficient = 6.71, *p*-value = 0.003. Participants with a bachelor’s degree have more positive attitudes as compared to those with an educational level lower than high school; coefficient = 7.23, *p*-value = 0.001 (Table 10).

## 4. Discussion

### 4.1. General Obesity

Obesity continues to be a pressing global health issue, contributing to rising rates of chronic diseases such as type 2 diabetes, cardiovascular disorders, and certain cancers. Bariatric surgery, as a viable intervention for severe obesity, offers a significant opportunity to mitigate these health burdens. This study sought to assess the knowledge and attitudes of the Saudi population regarding bariatric surgery, providing insight into barriers, misconceptions, and potential facilitators for adopting this treatment in the region [2]. The startlingly high rate of obesity in Saudi Arabia is indicative of a wider trend observed in many developed nations. Recent research indicates that nearly 50% of Saudi Arabian adults are obese, and rising rates among children and adolescents are also a cause for concern in the kingdom [10]. One effective way of reducing obesity when diet and exercise fail is bariatric surgery, which helps individuals achieve substantial weight loss by modifying the digestive system [14].

However, bariatric surgery remains a relatively new field not only for healthcare professionals but also for the general public. Therefore, this research was carried out to assess the awareness and attitudes of the Saudi population towards bariatric surgery.

### 4.2. Knowledge on and Attitudes Towards Obesity

Participants demonstrated a high level of knowledge about obesity, with a mean score of 4.08 (±0.60) out of 5. Most participants recognized obesity as a disease (41.9%) and identified key factors such as poor diet and lack of exercise as contributors. However, misconceptions remain, with only 2.6% attributing obesity solely to genetics. These results align with studies conducted in Al-Qassim Region and Buraydah City, which also reported high obesity knowledge levels among Saudi participants [19,20]. Despite this strong knowledge base, the relatively moderate awareness of bariatric surgery suggests a disconnect between understanding obesity as a disease and considering surgical interventions as a viable treatment option. This disconnect warrants further investigation in order to tailor public health education effectively.

The only factor associated with level of knowledge on obesity was monthly income level. The results showed that participants who earn less than SAR 5000 have less knowledge as compared to unemployed participants; coefficient = −0.223, *p*-value = 0.047. Participants who earn from SAR 5000 to 9999 have less knowledge as compared to unemployed participants; coefficient = −0.37, *p*-value = 0.010. Higher knowledge was seen among participants who had a monthly salary from SAR 10,000 to 20,000; coefficient = 1.58, *p*-value = 0.018. Also, a higher BMI was associated with higher knowledge; coefficient = 0.08, *p*-value = 0.010.

The observed disparity in knowledge about obesity based on monthly income levels highlights a significant socioeconomic factor influencing health literacy. A knowledge gap may result from lower-income participants having more urgent financial problems taking precedence over health education. Also, it is important to consider that people with lower earnings frequently have to make food choices based on budgetary restraints, which forces them to choose less nutritious but less expensive products. These people may regularly turn to processed foods and fast food, which are often more economical but higher in unhealthy fats, sugars, and sodium, to make ends meet. Thus, interventions should not only be to improve health literacy but also to support lower-income groups’ access to healthier, more affordable food options.

### 4.3. Factors Associated with Knowledge on Bariatric Surgery

Participants’ willingness to undergo bariatric surgery was low, with only 7.6% expressing readiness to consider the procedure. Knowledge about bariatric surgery was moderate, with a mean score of 3.28 (±1.46) out of 6. Most participants recognized the benefits of bariatric surgery, such as significant weight loss for morbidly obese individuals (48.9%), but many held misconceptions, including the belief that the procedure carries no risks (72.2%). These misconceptions highlight the need for targeted educational interventions to address safety concerns and promote informed decision-making. The results indicated a lower level of knowledge about bariatric surgery with a mean score of 3.28 (out of 6). The majority of the participants (48.9%) believe that bariatric surgeries contribute to significant weight loss in morbidly obese patients, while a relatively low percentage of the participants (3.5%) believe that bariatric surgeries should be illegal. A similar study conducted in Jeddah showed that when participants were asked about their knowledge of bariatric surgery, 31.7% of participants were not informed about bariatric surgery, and 21.5% of participants were poorly informed about it [21].

One of the notable findings of the present study is that it highlights four important factors associated with the level of knowledge about bariatrics surgery in society.

Firstly, the results show that females had higher knowledge about bariatric surgery than males. Female participants and those with higher BMI demonstrated greater knowledge about bariatric surgery, consistent with previous findings [17,22]. However, income level’s inconsistent association with knowledge suggests the need for more nuanced research to explore the relationship between socioeconomic status and health literacy. This gender disparity might be attributed to the fact that women are often more proactive in interacting with healthcare providers and requesting health information, especially when it comes to weight control and cosmetic procedures [22]. Furthermore, women’s body image and weight are frequently given more weight by society and cultural norms; as a result, some view bariatric surgery as both a medical intervention and a cosmetic operation to improve their looks [23]. The gender disparity in knowledge may reflect societal emphasis on women’s body image and greater healthcare engagement among women. Tailoring educational campaigns to these dynamics could enhance their effectiveness.

Secondly, a higher BMI was associated with higher knowledge. This result is similar to that of a study conducted in Saudi Arabia where participants with satisfactory knowledge scores had a higher mean BMI than those with unsatisfactory knowledge scores [17]. When diet and exercise alone are unable to result in noticeable weight loss, bariatric surgery frequently becomes a reasonable alternative for those who are severely obese. As a result, those with higher BMIs are more inclined to actively seek out alternative ways to manage their weight and enhance their health, which increases their knowledge about bariatric surgery [24].

Thirdly, participants who earn from SAR 10,000 to 20,000 have a higher knowledge on bariatric surgery as compared to unemployed participants. However, other studies all concluded that income level had no significant correlation with knowledge of obesity and bariatric surgery [17,22]. This reiterates the importance of conducting more research focusing on the correlation between income level and knowledge on bariatric surgery.

Lastly, participants with a high school education or above showed more positive attitudes than those without a high school diploma. A study conducted among adult Saudi showed that educational level is significantly correlated with knowledge of obesity, with people with higher educational levels correctly identifying obesity as a disease [17]. Furthermore, studies on physicians’ knowledge on and attitudes toward bariatric surgery in Saudi Arabia showed that physicians with higher education had positive attitudes toward bariatric surgery [18,25]. In general, opinions toward bariatric surgery can differ based on personal characteristics including socioeconomic status, gender, and educational level. It is crucial that healthcare professionals take these factors into account and clear up any misunderstandings or worries patients may have concerning bariatric surgery.

The optional questions of the present study provided additional insights, indicating varying opinions on healthcare insurance coverage: 65.8% wanted the surgery to be covered, while 34.2% did not think it should be covered. When physicians were asked about bariatric surgery affordability, 45.7% agreed that their obese patients cannot afford bariatric surgery [18].

### 4.4. Policy Implications and Recommendations

The findings from this study highlight the need for targeted public health campaigns to improve knowledge about bariatric surgery. Such campaigns should address common misconceptions about the procedure, including its risks and benefits, while promoting broader access through insurance coverage and subsidy programs for lower-income individuals. Expanding healthcare providers’ training on bariatric surgery could enhance referral rates and ensure accurate patient counseling. Policymakers should also consider integrating bariatric surgery education into broader obesity prevention and management strategies, leveraging gender-specific and culturally tailored approaches to engage different population groups effectively.

### 4.5. Limitations

This study has several limitations. The use of convenience sampling, combined with the inclusion of medical students, raises concerns about the generalizability of the results to the broader Saudi population. Medical students may have better access to health information, and thus, their over-representation could have skewed the results toward higher knowledge levels. Additionally, the achieved sample size, though sufficient for descriptive insights, was lower than the target, potentially limiting the power to detect smaller associations. Finally, the reliance on self-reported data may introduce recall or social desirability bias, while the lack of regional or nationality-specific data limits our ability to identify localized patterns. Future studies should aim for stratified random sampling and larger sample sizes to improve representativeness and statistical power. Including regional data could provide valuable insights into geographic disparities in awareness and attitudes.

## 5. Conclusions

In conclusion, this study highlights moderate awareness and mixed attitudes toward bariatric surgery among the Saudi population, with persistent misconceptions about its risks and benefits. Addressing these gaps through tailored educational initiatives and policy interventions could enhance the acceptance and accessibility of bariatric surgery. By aligning public health strategies with these findings, Saudi Arabia can make significant progress in addressing the obesity epidemic and improving health outcomes.

## Figures and Tables

**Table 1:** Characteristics of the participants (*n* = 313).

	*n*	%
Gender		
Male	166	53.0
Female	147	47.0
Marital status		
Single	126	40.3
Married	187	59.7
Monthly income level		
Unemployed	78	24.9
SAR < 5000	47	15.0
SAR 5000–9999	36	11.5
SAR 10,000–20,000	73	23.3
SAR > 20,000	79	25.2
Educational level		
Lower than high school	3	1.0
High school	82	26.2
Bachelor’s degree	159	50.8
Post-graduate degree	69	22.0
	Mean	SD
Height (in cm)	168.01	9.03
Weight (in kg)	76.61	17.8
BMI (kg/m^2^)	26.98	5.36

Note: USD 1 = SAR 3.75 for reference; SAR 5000 = USD~1331.81. The income reported here is monthly income.

**Table 2:** Participants’ willingness to undergo bariatric surgery.

	*n*	%
If you haven’t undergone bariatric surgery, would you take it?		
No	223	71.20%
Yes	63	20.10%
Doesn’t apply	27	8.60%

**Table 3:** Knowledge about obesity and bariatric surgery.

Knowledge About Obesity	No	Yes	I Don’t Know
Obesity is an increased deposition of fat in the body	9 (2.9%)	290 (92.7%)	14 (4.5%)
A healthy diet reduces obesity	6 (1.9%)	306 (97.8%)	1 (0.3%)
Excessive eating leads to obesity	19 (6.1%)	292 (93.3%)	2 (0.6%)
Exercise is the best way to lose weight	85 (27.2%)	222 (70.9%)	6 (1.9%)
People who are overweight are more susceptible to chronic illnesses such as type 2 diabetes and hypertension	1 (0.3%)	306 (97.8%)	6 (1.9%)
Mean score (mean ± SD)	4.08 ± 0.60		
Knowledge about bariatric surgery			
Bariatric surgery is indicated solely for excessively obese patients (when BMI [body mass index] is greater than 40)	35 (11.2%)	197 (62.9%)	81 (25.9%)
Patients undergoing bariatric surgery require lifetime vitamin supplementation	42 (13.4%)	160 (51.1%)	111 (35.5%)
Bariatric surgery increases life expectancy	39 (12.5%)	146 (46.6%)	128 (40.9%)
Surgery is the best way to get rid of obesity	231 (73.8%)	49 (15.7%)	33 (10.5%)
There are no risks or complications in bariatric surgery procedures	226 (72.2%)	26 (8.3%)	61 (19.5%)
Bariatric surgery reduces the risk of developing cancer	71 (22.7%)	67 (21.4%)	175 (55.9%)
Mean score (mean ± SD)	3.28 ± 1.46		

Note: The total score (mean ± SD) represents the cumulative knowledge score for each domain, calculated by summing the correct responses to knowledge-related questions. Scores range from 0 to 5 for knowledge of obesity and 0 to 6 for knowledge of bariatric surgery.

**Table 4:** Attitudes regarding obesity and bariatric surgery.

	Strongly Disagree	Disagree	Neutral	Agree	Strongly Agree
Attitudes regarding obesity					
I believe that obesity is a disease.	20 (6.4%)	21 (6.7%)	68 (21.7%)	73 (23.3%)	131 (41.9%)
I believe that obesity is caused solely by genetics.	111 (35.5%)	62 (19.8%)	103 (32.9%)	29 (9.3%)	8 (2.6%)
I believe that obesity is solely caused by diet.	133 (42.5%)	53 (16.9%)	75 (24%)	36 (11.5%)	16 (5.1%)
I believe that mental illnesses such as anxiety can lead to obesity.	11 (3.5%)	21 (6.7%)	82 (26.2%)	103 (32.9%)	96 (30.7%)
I believe that aging causes obesity.	44 (14.1%)	68 (21.7%)	113 (36.1%)	62 (19.8%)	26 (8.3%)
Mean score (mean ± SD)	16.58 ± 2.68				
Attitudes regarding bariatric surgeries					
I believe that bariatric surgeries contribute to significant weight loss in morbidly obese patients.	8 (2.6%)	9 (2.9%)	43 (13.7%)	100 (31.9%)	153 (48.9%)
I believe that bariatric surgeries should be illegal.	145 (46.3%)	68 (21.7%)	75 (24%)	14 (4.5%)	11 (3.5%)
Bariatric surgery is my first choice to lose weight.	187 (59.7%)	52 (16.6%)	49 (15.7%)	10 (3.2%)	15 (4.8%)
I believe that bariatric surgery is the easiest and quickest solution to obesity.	72 (23%)	51 (16.3%)	83 (26.5%)	57 (18.2%)	50 (16%)
I believe that bariatric surgery can lead to death.	35 (11.2%)	60 (19.2%)	124 (39.6%)	62 (19.8%)	32 (10.2%)
I believe that bariatric surgery is cosmetic.	78 (24.9%)	75 (24%)	90 (28.8%)	45 (14.4%)	25 (8%)
Mean score (mean ± SD)	19.33 ± 3.72				

The total score (mean ± SD) reflects the overall attitude score, derived by summing the coded responses for attitude-related questions. Higher scores indicate more positive attitudes toward the respective domains (obesity and bariatric surgery).

**Table 5:** Correlation between knowledge about obesity and bariatric surgery.

		Knowledge About Bariatric Surgery
Knowledge about obesity	Pearson correlation	0.137
	*p*-value	0.015

**Table 6:** Correlation between attitudes regarding obesity and bariatric surgery.

		Attitudes Regarding Bariatric Surgery
Attitudes regarding obesity	Pearson correlation	0.194
	*p*-value	0.001

**Table 7:** Factors associated with knowledge about obesity.

	Coefficient	*p*-Value	95% C.I.	
			Lower Bound	Upper Bound
Age	−0.002	0.592	−0.009	0.005
Gender				
Male	Ref			
Female	0.128	0.054	−0.002	0.257
Marital status				
Single	Ref			
Married	0.009	0.944	−0.247	0.266
BMI	0.000	0.954	−0.013	0.012
Monthly income level				
Unemployed	Ref			
SAR < 5000	−0.223	0.047	−0.443	−0.003
SAR 5000–9999	−0.376	0.010	−0.663	−0.089
SAR 10,000–20,000	−0.184	0.184	−0.455	0.088
SAR > 20,000	−0.092	0.54	−0.389	0.204
Educational level				
Lower than high school	Ref			
High school	−0.286	0.407	−0.963	0.391
Bachelor’s degree	−0.213	0.53	−0.882	0.455
Post-graduate degree	−0.149	0.666	−0.828	0.530

Ref = reference category.

**Table 8:** Factors associated with knowledge regarding obesity.

	Coefficient	*p*-Value	95% C.I.	
			Lower Bound	Upper Bound
Age	−0.02	0.36	−0.05	0.02
Gender				
Male	Ref			
Female	0.43	0.182	−0.20	1.05
Marital status				
Single	Ref			
Married	−0.72	0.251	−1.96	0.51
BMI	0.08	0.01	0.02	0.14
Monthly income level				
Unemployed	Ref			
SAR < 5000	1.13	0.037	0.07	2.19
SAR 5000–9999	1.34	0.058	−0.04	2.73
SAR 10,000–20,000	1.58	0.018	0.27	2.89
SAR > 20,000	1.05	0.149	−0.38	2.48
Educational level				
Lower than high school	Ref			
High school	1.46	0.381	−1.81	4.73
Bachelor’s degree	1.46	0.372	−1.76	4.69
Post-graduate degree	1.19	0.476	−2.09	4.47

Ref = reference.

**Table 9:** Factors associated with knowledge about bariatric surgery.

	Coefficient	*p*-Value	95% C.I.	
			Lower Bound	Upper Bound
Age	−0.01	0.244	−0.03	0.01
Gender				
Male	Ref			
Female	0.80	<0.001	0.48	1.12
Marital status				
Single	1.00			
Married	0.43	0.181	−0.20	1.07
BMI	0.04	0.004	0.01	0.07
Monthly income level				
Unemployed	Ref			
SAR < 5000	0.04	0.878	−0.50	0.59
SAR 5000–9999	−0.22	0.536	−0.94	0.49
SAR 10,000–20,00	−0.44	0.200	−1.11	0.23
SAR > 20,000	0.13	0.736	−0.61	0.86
Educational level				
Lower than high school	Ref			
High school	−0.69	0.422	−2.37	0.99
Bachelor’s degree	−0.30	0.723	−1.96	1.36
Post-graduate degree	0.22	0.794	−1.46	1.91

Ref = reference.

**Table 10:** Factors associated with attitudes regarding bariatric surgery.

	Coefficient	*p*-Value	95% C.I.	
			Lower Bound	Upper Bound
Age	0.03	0.169	−0.01	0.08
Gender				
Male	Ref			
Female	0.52	0.228	−0.32	1.36
Marital status				
Single	Ref			
Married	−0.96	0.256	−2.62	0.70
BMI	0.04	0.359	−0.04	0.12
Monthly income level				
Unemployed	Ref			
SAR < 5000	2.04	0.005	0.61	3.47
SAR 5000–9999	1.44	0.129	−0.42	3.30
SAR 10,000–20,000	2.01	0.025	0.25	3.77
SAR > 20,000	1.86	0.058	−0.06	3.78
Educational level				
Lower than high school	1.00			
High school	6.71	0.003	2.32	11.10
Bachelor’s degree	7.23	0.001	2.90	11.56
Post-graduate degree	7.33	0.001	2.93	11.73

Ref = reference.

## Data Availability

The data used to support the findings of this study are available upon request from the corresponding author.
